# The biochemical value of urinary metalloproteinases 3 and 9 in diagnosis and prognosis of bladder cancer in Egypt

**DOI:** 10.1186/s12929-014-0072-4

**Published:** 2014-08-19

**Authors:** Fathia El-Sharkawi, Mahmoud El Sabah, Zeinab Hassan, Hussein Khaled

**Affiliations:** 1 Department of Biochemistry and Molecular Biology, Faculty of Pharmacy, Helwan University, Cairo, Ein helwan, Egypt; 2 Department of Biochemistry, Faculty of Pharmacy, Future University, Cairo, El tagamu El khames, Egypt; 3 Department of Medical Oncology, National Cancer Institute, Cairo University, Cairo, Kasr el Eini, Egypt

**Keywords:** Bladder cancer, Schistosoma, MMP3,MMP9

## Abstract

**Background:**

Matrix metalloproteinases (MMPs) have long been associated with cancer-cell invasion and metastasis. Few studies are available that describe this association with bladder cancer either related or unrelated to schistosoma infection.

Evaluating the urinary levels of MMP3 and MMP9 as diagnostic and prognostic biomarkers in different stages of schistosomal and non schistosomal bladder cancer was the aim of the present study.

Urine samples were collected from 70 patients with schistosomal and non schistosomal bladder cancer at early and advanced stages and also from12 healthy volunteers as controls. Urinary levels of MMP-3 and MMP-9 was measured by ELISA technique. Sensitivity and specificity of both markers were determined.

**Results:**

Urinary levels of both MMP-3 and MMP-9 were significantly elevated in all bladder cancer patients compared with controls. MMP-3 started to elevate in early stages of schistosomal bladder cancer ( 0.173 ng/ml) and non-schistosomal bladder cancer patients (0.308 ng/ml) compared to control (0.016 ng/ml) and remained elevated in advanced stages (0.166, 0.235 ng/ml) of both types of bladder cancer patients. In contrast, MMP-9 showed a significant elevation in advanced stages only of both schistosomal and non schistosomal bladder cancer patients (10.33, 21.22 ng/ml) compared to control (0.409 ng/ml) and this elevation of both markers was much higher in non schistosomal bladder cancer. Both Metalloproteinases were specific for the diagnosis of the disease but MMP-3 was more sensitive and this sensitivity was evident in the early stage (84.85% for MMP3, 27.28% for MMP9).

**Conclusions:**

MMP3 may be the recommended urinary metalloproteinases as early diagnostic biomarker in the early stages of both types of bladder cancer although both MMP9 and MMP3 can be used in the diagnosis of advanced stages. Further studies are required on large number of urine samples to confirm these results.

## Background

Bladder cancer is an important public health problem and is considered the fourth most common malignant neoplasm in men and the eighth in women where more than 70,530 new cases were estimated in 2010 accounting for more than 14,680 deaths in USA [1]. In Egypt it is the first site of incidence for males accounting for 17% of all cancer cases; while in females it accounts for 5%, ranking first among male cancer with a male/female ratio of 3.5:1 [2]. Schistosomiasis has been endemic in Egypt at least since the time of the ancient Pharaohs, as indicated by the presence of calcified ova in the Egyptian mummies [3]. The association of bladder cancer with schistosomiasis seems to be related to the endemicity of the parasite, and is considered the main etiologic factor [4].

Matrix metalloproteinases (MMPs) are a large family of calcium-dependent zinc-containing endopeptidases that are involved in extracellular matrix (ECM) degradation. MMPs are capable of degrading extracellular molecules and a number of bioactive molecules. Twenty four related genes have been identified in humans, which can be organized into six groups based on domain organization and substrate preference. MMPs play a central role in cell proliferation, migration, differentiation, angiogenesis, apoptosis and host defenses. It has been reported that deregulation of MMPs has been implicated in many diseases including arthritis, chronic ulcers, encephalomyelitis and cancer [5].

The gold standard for bladder cancer screening is still urine cytology, as it is non-invasive, safe and inexpensive. Although it is highly specific, the results are not reproducible and the interpretation is highly dependent on the skill of the cytologist [6]. This calls for searching other markers for screening of bladder cancer which should be specific, sensitive, reproducible, non-invasive and done at an acceptable cost.

So the aim of the present study was to evaluate the urinary levels of MMP3 and MMP9 in different stages of schistosomal and non-schistosomal bladder cancer in Egyptian patients.

## Methods

A prospective analysis was performed on 70 bladder cancer Egyptian patients who have attended at the cystoscopy unit of the National Cancer Institute, Cairo University, during the period from May 2010 to March 2012 and 12 healthy people with no previous history of any urological disorders as a control group.

All subjects signed an informed written consent for participation and the study was approved by the research ethics committee of the General Organization for Teaching Hospitals and Institutes. Patients demographic data and medical history were obtained from hospital files. Pathological grading of cases was based on the World Health Organization’s classification of urothelialneoplasms [7]. Staging of bladder cancer was according to the classification of Union for International Cancer Control (UICC TNM) [8]. Treatment of the 70 patients included in the study followed the guidelines adopted at the National Cancer Institute, Cairo University where all T1 cases were subjected to transuretheral resection and local BCG, while radical cystectomy was done for those having T2-3 disease stage +/−pelvic radiotherapy and/or adjuvant chemotherapy. T4 cases received neoadjuvant chemotherapy to be followed by local therapy.

### Experimental samples

Patients were classified into:

1. Schistosomal bladder cancer group: 36patients (33 males and 3 females with a mean age of 62.7 and a range of 38–84 years). Seventeen patients (15 males and 2 females) presented with early stages of the disease (T1-T2) and 19 patients (18 males and one female) had locally advanced disease stages (T3-T4).

2. Non schistosomal bladder cancer group: 34 patients (19 males and15 females; with a mean age of 59.8 and a range of: 44–73 years). Sixteen patients presented with early stages and 18 patients with locally advanced stages.

The 12 healthy people that have served as a control group were matched with sex and age with the bladder cancer group (Table 1).

**Table 1:** Demographic data of the bladder cancer cases and the control group

**Demographics**	**Schistosomal group**	**Non-schistosomal group**	**Total**	**controls**
Age: Mean	62.77	59.8	61.3	50.5
(years) Range	38-84	44-73	32-84	29-66
Sex: Male	33	19	52	7
Female	3	15	18	5
Disease Stage: T 1–2	17	16	33	12
T 3-4	19	18	37	
Total	36	34	70	12

Freshly voided urine was collected from all patients and controls; samples were centrifuged at 1000 rpm and separated as urine and urine sediment. All samples were taken at the time of diagnosis before receiving any treatment.

### ELISA

Urinary MMP9 and MMP3 were measured, using a commercially available ELISA kits provided by R&D Systems, Inc.614 McKinley, United States of America with catalog number DMP900 and from Cusabio with calaloge number of CSB-E04677h, according to manufacturer instructions.

Two microplates of 96 wells each has been pre-coated with monoclonal antibodies specific for MMP-9 and MMP3. Standards and urine samples were pipetted into the wells of each microplate. After washing away unbounded substances, an enzyme-polyclonal antibody specific for each MMp-9 and MMP3 was added to the wells of each plate. Following washing to remove unbound antibody- enzyme reagent, a substrate solution was added to the wells of each plate and a color was developed in proportion to the amount of MMP-9and MMP3 bounded in the initial step. The intensity of the color was measured at specific wave length. Standard curves were plotted using standard concentrations on X axis and their optical densities on Y axis.

The concentrations of MMP-9 and MMP3 in urine samples were determined by comparing the optical densities (OD) of the samples to the standard curves.

### Statistical analysis

The SPSS software system for Windows (version 12.0; SPSS, Chicago, IL, USA) was utilized for statistical analysis. A statistical computer program (GraphPadInstat) was used to test the significance of the difference between patients groups.

One-way Analysis of Variance (ANOVA) for parametric data was used to analyze more than two sets of data.ROC curves generated from ELISA data were used to measure the diagnostic accuracy of the measured parameters. Data was presented as mean value ± SE.

## Results

Data was presented as mean value ± SE of the bladder cancer patients compared with that of healthy control people.

### Urinary matrix metalloproteinases-9 (MMP9)

There was a significant increase of the mean value of urinary MMP9 level for the whole 70 bladder cancer cases (7.99 ng/ml) when compared to that of the control group (0. 409 ng/ml) with a P value of < 0.001. In schistosomal bladder cancer group (36 cases),the level of MMP9 was elevated significantly to 5.25 ng/ml than that of controls (P < 0.001).Also this increase in MMP9 was shown in patients with non-schistosomal bladder cancer (34 cases, 10.73 ng/ml)compared to control level (P < 0.001). When comparing the level of MMP9 in schistosomal and non-schistosomal bladder cancer groups; there was also a significant elevation of MMP9 in non-schistosomal bladder cancer group when compared to the schistosomal bladder cancer group.

**Figure 1:**
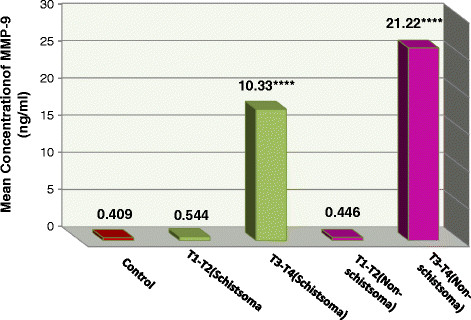
**Mean concentration of urinary MMP9 in schistosomal and non schistosomal bladder cancer.** The values were expressed as mean. ****P <0.0001 compared with control using t test for unpaired data.

There was a variation in the level of MMP9 between the patients in different stages of bladder cancer compared to control; at stages T1and T2, this variation was not significant neither in schistosomal bladder cancer (0.544 ng/ml) nor in non-schistosomal bladder cancer patients (0.446 ng/ml). In contrast MMP9 was significantly highly increased in stages T3 and T4 of both schistosomal bladder cancer (10.33 ng/ml) and non-schistosomal bladder cancer patients (21.22 ng/ml) (P < 0.0001) and this increase was much higher in non-schistosomal bladder cancer than in schistosomal bladder cancer patients (Table 2) and (Figure 1).

**Table 2:** Mean urinary levels of MMP9 and MMP3 for schistosomal and non-schistosomal bladder cancer patients

**A. (MMP9)**			
**Schisto/Stage**	**T 1-T2 stage**	**T 3-T4 stage**	**Total**
**Non-Schistosomal cases**	**0.446**	21.22****	**10.73**
**Schistosomal cases**	**0.544**	10.33****	**5.25**
**Total**	**0.495**	**15.78**	**7.99**
**B. (MMP3)**			
**Schisto/Stage**	**T 1-T2 stage**	**T 3-T4 stage**	**Total**
**Non-Schistosomal cases**	0.308***	0.235***	**0.269**
**Schistosomal cases**	0.173***	0.166***	**0.169**
**Total**	**0.241**	**0.201**	**0.219**

A. Mean value of control group 0.409 ng/ml.

B. Mean value of control group 0.016 ng/ml.

The values were expressed as mean. ****P < 0.0001, ***P <0.001 compared with control using t test for unpaired data.

### Urinary matrix metalloproteinases-3(MMP3)

There was a significant difference between the mean level of urinaryMMP3 of the control group (0.016 ng/ml) and that of the 70 bladder cancer cases (0.219 ng/ml, P < 0.001). MMP3 was elevated significantly in both schistosomal bladder cancer (0.169 ng/ml) and non schistosomal bladder cancer groups (0.269 ng/ml) compared to the control group (P < 0,001); with more significant increase in non schistosomal bladder cancer than schistosomal bladder cancer patients.

In contrast to MMP9 it was shown that the level of MMP3 started to elevate significantly ((P <0.001) in patients with T1and T2 stages (0.241 ng/ml) and remains significantly elevated at more advanced T3and T4 stages (0.201 ng/ml), when compared to controls (0.016 ng/ml).

**Figure 2:**
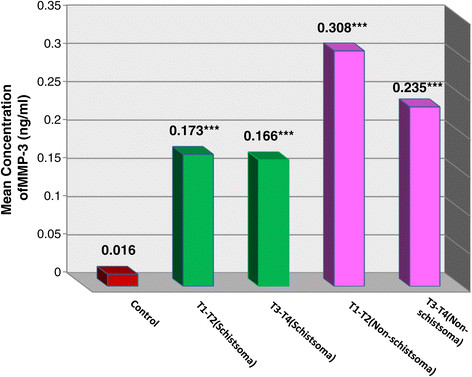
**Mean concentration of urinary MMP3 in schistosomal and non schistosomal bladder cancer.** The values were expressed as mean. ***P <0.001 compared with control using t test for unpaired data.

The significant elevation of MMP3 was much higher in the early stages of non schistosomal bladder cancer (0.308 ng/ml, P <0.001) than schistosomal bladder cancer (0.173 ng/ml, P <0.001). In more advanced stages of both types of bladder cancer, MMP3 was similar to MMP9 in its significant elevation than controls both in schistosomal bladder cancer (0.166 ng/ml) and non schistosomal bladder cancer groups (0.235 ng/ml) and this elevation was higher in non schistosomal bladder cancer patients (Table 2) and ( Figure 2).

### Diagnostic accuracy of the investigated biomarkers

ROC curves generated from ELISA were used for measuring the accuracy of both MMP9 and MMP3 at different stages of bladder cancer. It was found that both metalloproteinases were specific for diagnosis of the disease being 100% for MMP9 and 92.31% for MMP3 (91.67% for early and 100% for late stages). The sensitivity was variable (Table 3); MMP3 was more sensitive being 86.42% while it was only 64.29% for MMP9. This difference in sensitivity between the 2 markers was more evident in the early stages (84.85% for MMP3 compared to 27.28% for MMP9).

**Table 3:** Sensitivity and specificity of MMP9 and MMP3 for early and advanced stages of bladder cancer patients

**A. (MMP9)**		
**Stage/parameter %**	**Sensitivity**	**Specificity**
**Early stages**	**27.28**	**100**
**Advanced stages**	**97.3**	**100**
**Total**	**64.29**	**100**
**B. (MMP3)**		
**Stage/parameter %**	**Sensitivity**	**Specificity**
**Early stages**	**84.85**	**91.67**
**Advanced stages**	**89.19**	**100**
**Total**	**86.42**	**92.31**

## Discussion

The majority of the biomarker studies in patients with bladder cancer have been focused on urine [9]. The association between the urinary levels of MMP9 and risks of bladder cancer as well as stage and grade of disease was reported in different studies [10], but few studies are available for MMP3 levels in bladder cancer both schistosoma or non-schistosoma related.

Metalloproteinases are a family of proteolytic enzymes that degrade all of the main components of the extracellular matrix and basement membrane [11]. They are associated with the cell surface and facilitate the invasion of cancer cells through epithelial cell layers and into nearby stroma and blood vessels, and generally they are over expressed in human tumors [12]. Also they are known to participate in angiogenesis and tumor growth particularly gelatinases (gelatinase A, MMP2, gelatinase B, and MMP9) [13].

In the current study, urinary MMP9 was not increased in superficial stages of both types of bladder cancer either related or unrelated to schistosoma infection. Durkan et al. reported that there was no significant difference in urinary MMP9 concentrations between patients with bladder cancer and patients with benign urological disorders; while concentrations of urinary MMP9 were significantly higher in urine samples from patients with T3–T4 tumors compared with samples from patients with early tumors [14]. Also Davies et al. reported that levels of MMP9 and activated MMP2 were higher in invasive tumors than in superficial ones [15]. The results of these two studies are in agreement with our study. Moreover, the current study is distinctive in reporting the role of urinary MMP3 in bladder cancer, whether of schistosomal or non-schistosomal origin.

MMP3 is expressed by fibroblastic cells and by normal and transformed squamous epithelial cells [16]; where as MMP9 is strongly expressed in intravascular and tissue-infiltrating leucocytes [17]. Also MMP3 is one of the most effective activators for MMP9 [18]. This may explain the increase in urinary levels of MMP3 rather than MMP9 in the early stages of both schistosomal and non schistosomal bladder cancer patients.

In the present study the urinary concentrations of both MMP3 and MMP9 were much higher in non schistosomal bladder cancer patients than in schistosomal ones and these findings are different from those of Fouda et al., who reported that there was no significant increase in MMP-9 activity in bilharzial related TCC type compared to non bilharzial one [19]. This controversy may be due to several issues like the difference in the used methodology between the two studies since Fouda et al., used zymographic analysis while ELISA technique was used in the current study, also Fouda et al., study didn’t identify the stages of the bladder cancer disease ,it depends on type of cancer whether TCC or SCC but in our study the selection of the patients was according to their disease stage whether they were at early (T1-T2) or advanced (T3-T4) stages which is considered to be more valuable and more accurate. Further more Fouda et al., study included 50 patients and this difference in number of patients in each study may affect the obtained results. We agree with Fouda et al., that more studies are needed in the future, but from our point of view large number of schistosomal and non schistosomal patients at different stages of the bladder cancer and with TCC and SCC must be included.

## Conclusions

So in conclusion, measurements of urinary levels of MMP3 and MMP9 may aid in the diagnosis of both schistosomal and non schistosomal bladder cancer at different stages. While MMP3 may be used as diagnostic biomarker in early stages; MMP9 may help as diagnostic biomarker only in advanced stages. However, further studies are required on large number of urine samples to confirm these results especially the role of MMP3 in the diagnosis of early stages of schistosomal and non schistosomal bladder cancer.

## Competing interests

The authors declare that they have no competing interests.

## Authors’ contribution

FEl: Constructed the idea of the experiment, participated in the analysis of the data and interpretation of results, and wrote the manuscript. MEl: Collected the patient’s samples, carried out the practical work and statistical analysis. ZH: Participated in the revision of results and statistical analysis. HK: Constructed the idea and the design of the work, participated in the revision of the results and statistical analysis, reviewed the manuscript.
